# Supplementary material for: Immunomodulatory Effects of Non-Thermal Plasma in a Model for Latent HIV-1 Infection: Implications for an HIV-1-Specific Immunotherapy
Source: Biomedicines. 2023 Jan 3;11(1):122. doi: 10.3390/biomedicines11010122 (PMC9856147; doi:10.3390/biomedicines11010122)
Supplement: Supplementary file 1 [file biomedicines-11-00122-s001.zip › biomedicines-2039019-supplementary.pdf]

biomedicines

MDPI

Article

Immunomodulatory effects of non-thermal plasma in a model for latent HIV-1 infection: Implications for an HIV-1-specific immunotherapy

Hager Mohamed, Rachel Berman, Jennifer Connors, Elias K. Haddad, Vandana Miller, Michael R. Nonnemacher, Will Dampier, Brian Wigdahl, and Fred C. Krebs

Supplementary Materials

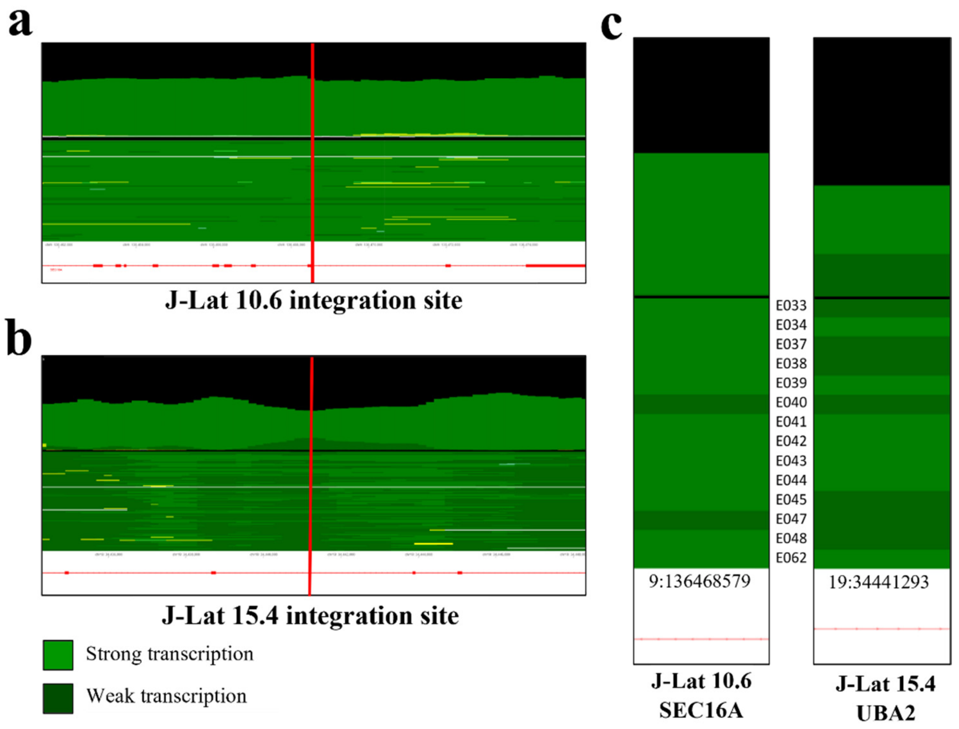

Supplementary Figure S1. Chromatin states around the HIV-1 provirus integration sites differ between J-Lat cell line clones. Visualization of the chromatin state in a 5 kb window around the integration sites of (**a**) J-Lat clone 10.6 in an intron of SEC16A and (**b**) J-Lat clone 15.4 in an intron of UBA2. (**c**) Visualization of the chromatin state at a single base resolution for each clone. The bottom portion (green portion) depicts the individual chromatin state per sample and the top (above the black line) is the expected state observed for the region. The state of transcription at the integration site is annotated as strong (3.854-fold enrichment for the observed feature) for clone 10.6 and both strong and weak (1.859- and 1.189-fold enrichment, respectively) for clone 15.4. Visualization of the chromatin state was obtained using the Epilogos browser, which draws on analyses of transcription in primary blood and T lymphocytes from the Epigenome Roadmap consortium: T lymphocytes from cord blood (E033), T lymphocytes from peripheral blood (E034), T helper cells from peripheral blood (E037, E040), T helper naive cells from peripheral blood (E038, E039), T helper cells PMA-I stimulated (E041), T helper 17 cells PMA-I stimulated (E042), T helper cells from peripheral blood (E043), T regulatory cells from peripheral blood (E044), Effector/memory T lymphocytes enriched from peripheral blood (E045), CD8+ naïve cells from peripheral blood (E047), CD8+ memory cells from peripheral blood (E048), and mononuclear cells from peripheral blood (E062).
